# Tailored treatment of female indirect inguinal hernias by using single-port laparoscopic percutaneous internal ring suture: a comparison between children and adults

**DOI:** 10.1007/s10029-024-03055-3

**Published:** 2024-05-22

**Authors:** S.‑H. Wang, C.-Y. Lee, W.-C. Hsieh, J.-B. Yen, I.-M. Tseng, C.-H. Wong, D.-R. Ho

**Affiliations:** 1 Department of Pediatric Surgery, Chang-Gung Memorial Hospital, Chiayi, 61363 Taiwan; 2 School of Medicine, College of Medicine, Chang-Gung University, 333, Taoyuan, Taiwan; 3 Department of Obstetrics and Gynecology, Chang-Gung Memorial Hospital, Chiayi, 61363 Taiwan; 4 Department of Nursing, Chang-Gung University of Science and Technology, Chiayi Campus, Chiayi, 61363 Taiwan; 5 Department of Rehabilitation, Chang-Gung Memorial Hospital, Chiayi, 61363 Taiwan; 6 Department of Pediatrics, Chang-Gung Memorial Hospital, Chiayi, 61363 Taiwan; 7 Department of Surgery, Chang-Gung Memorial Hospital, Chiayi, 61363 Taiwan; 8 Department of Anesthesiology, Chang-Gung Memorial Hospital, Chiayi, 61363 Taiwan; 9 School of Medicine, National Tsing Hua University, Hsinchu, 300044 Taiwan; 10 Department of Urology, Chang-Gung Memorial Hospital, Chiayi, 61363 Taiwan

**Keywords:** Inguinal hernia, Indirect type, Percutaneous internal ring suture, Female

## Abstract

**Purpose:**

To compare the outcome of indirect inguinal hernias repaired by using single-port laparoscopic percutaneous internal ring suture (SPIRS) between the pediatric and adult females.

**Methods:**

The medical records of females who were clinically assessed to have inguinal hernia from Oct. 2016 to May 2022 were reviewed. Patients who received laparoscopy for the diagnosis of the hernia type and customized treatment according to their hernia type were included, while those who chose other operation methods initially were excluded. The patients were divided into the adult and pediatric groups based on their age. The demographic characteristics, hernia types, operation durations, and outcomes were analyzed between these two groups.

**Results:**

A total of 65 adults and 60 children were included in this study. The median age was 38 years. (range: 23–88) for group A and 3 years (range: 0.1–16) for group P. Indirect hernias were present in 85% of adults and 100% of children. All the indirect hernias were repaired by SPIRS uneventfully. Incidence of contralateral patent processus vaginalis was 24% in adults and 50% in children (p = 0.016). The average operation time was 22/46 min (one/two sides) for the adults and 9/15 min (one/two sides) for the pediatrics (*p* < *0.010 for both*). The overall complication rates were 5.4% and 3.3% for the adult and pediatric group respectively (*p* = *0.106*). No recurrence was observed in the pediatric group, but two adults experienced recurrence and another had chronic postoperative inguinal pain, necessitating reoperation. The mean follow-up period was 38.6 ± 15.4 months for adults and 42.8 ± 18.9 months for children *(p* = *0.198*).

**Conclusion:**

Our results support that the pathogenesis of indirect inguinal hernia for the female adults is due to the non-obliteration of a congenital processus vaginalis. Tailored treatment of the female IIH by using single-port laparoscopic percutaneous internal ring suture may be an alternative for the management of female IHs.

## Introduction

As almost all of the pediatric inguinal hernias (IHs) are indirect type (IIH), laparoscopic percutaneous internal ring suture (PIRS) had been well developed and proved to be an effective and safe method for the repair of IH in the pediatric and young adolescent group since Dr. Patkowski first introduced this technique [1–5]. The principle of PIRS was to close the internal ring opening (IRO) at the preperitoneal level using a nonabsorbable suture with the assistance of laparoscopy.

Until now, the etiology of IIH for the adults remains ambiguous. However, a persistent nonobliteration of processus vaginalis (PV) from the infantile period is believed to be the most important factor contributing to the development of an adult IIH [6, 7]. Although previous literature has shown that most of the female IHs are indirect type [8, 9], post-surgical reoperation rates in females have been observed to be as high as 5.2% [10, 11]. A large-scale cohort study from Swedish Hernia Register also revealed that the standard repair techniques for males are not really suitable for females [9].

To improve the treatment outcomes of female IHs, our team previously proposed a strategy that involved identifying the hernia type using laparoscopy after general anesthesia, and then performing hernia repair based on the subtype [12]. In this study, we aim to compare the characteristics and outcomes of this procedure between young girls and adult females with IHs.

## Methods

The study received approval from the Ethical Review Board of Chang Gung Memorial Hospital. Medical records of the female patients who were clinically diagnosed to have IHs at our institute were reviewed. Only patients who received laparoscopic classification and tailoring treatment of their IHs were included in this study. Those opting for alternative initial repair methods were excluded. Patients were categorized as group P if aged < 20 years and group A if aged ≥ 20 years. 20 years was chosen because this is the age of adulthood legally.

The surgery was performed as we previously described [12]. Under general anesthesia, the patient was put in a supine position. By grasping and elevating the bilateral abdominal wall, a 3-mm sharpheaded trocar was inserted directly into the abdominal cavity via umbilicus, followed by a 3-mm zerodegree scope to confirm the successful trocar placement. Pneumoperitoneum was then established with intra abdominal pressures set at 15 mmHg for adults and 6–12 mmHg for children. The subtype of an IH was easily identified by viewing the posterior abdominal wall laparoscopically. To customize the treatment, an SPIRS would be carried out if IIH encountered, while mesh repair be performed for the other types (eg. direct, mixed, or femoral) of IH. If there was no abdominal wall defect found under laparoscopy, the inguinal region would be explored by open method.

### Surgical technique of SPIRS

The procedure for both groups were the same except a Gauze18 needle was used for children while a Gauze17 epidural needle (“B. Braun Melsungen AG”) was used for adults.

**Fig. 1:**
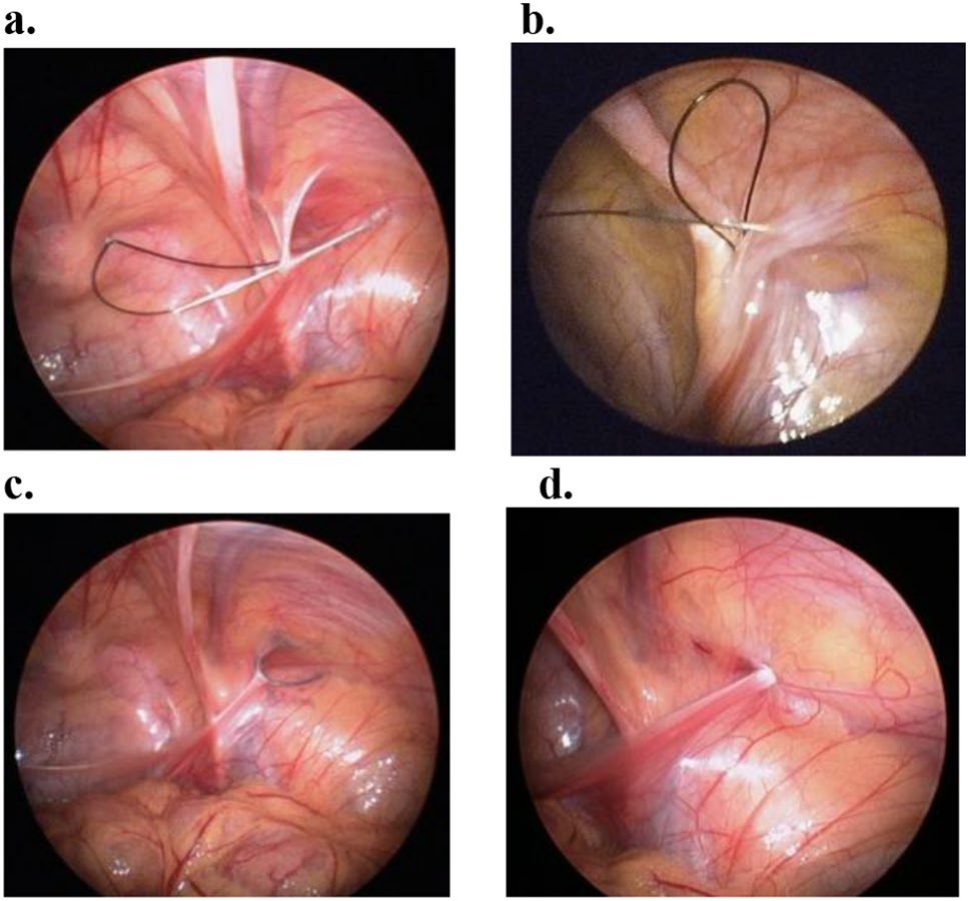
Key Procedures of SPIRS for Female Indirect Inguinal Hernia Repair. **a** The needle with a 2–0 Nylon was thrust into the abdominal cavity after passing the lower portion of internal ring opening (IRO). **b** A second 2–0 Nylon was introduced to surround the upper portion of IRO and came out from the loop of the first thread. **c** The IRO of hernia sac, including the round ligament, was encircled by the 2nd thread at the preperitoneal level. **d** The IRO was closed by tying the 2nd thread extracorporeally

The procedure can be broken down as follows:

1. The needle carrying a 2–0 Nylon thread was pierced into the abdominal wall and advanced around the lower hemi-circumference of the IRO, then came out into the abdominal cavity (Fig. 1a).
2. After needle removal, a loop of thread remains in the abdominal space.
3. A second 2–0 Nylon thread was introduced via the same punctured site, directed to surround the upper hemi-circumference of IRO at the preperitoneal level, and came out from the loop of the first thread (Fig. 1b).
4. After removing the needle, the 1st looping thread was pulled out of the skin, carrying the 2nd thread out of skin simultaneously, resulting in encirclement of the IRO by the 2nd thread at the preperitoneal level (Fig. 1c).
5. The IRO was then closed by tying the 2nd thread extracorporeally (Fig. 1d).
  *Tips: Before closure, the intra abdominal pressure was decreased to 4-5 mmHg to facilitate the extracorporeal tying and avoid stitch tear. After closure, we routinely resumed the intra abdominal pressure to 15 mmHg to confirm the security *[12]*.*
6. If present, the contralateral processus vaginalis (PV) is examined and similarly sealed using SPIRS.
  All the operations were performed by one surgeon. The postoperative pain was managed with the oral nonsteroidal anti-inflammatory agent, diclofenac 25 mg for adults and ibuprofen 5 mg/kg for children, three times a day for 3 days. Postoperative assessments, including wound pain, daily activity resumption, cosmetic satisfaction, and recurrence, were conducted at our outpatient clinic. The characteristics and outcomes of patients who have IIHs were compared between these two groups.

### Statistical analysis

The quantitative variables were presented as the mean ± standard deviation and qualitative variables as the frequency and percentage. Because of the normal distribution of the data, the independent t-test was used to assess the difference in means. The χ2 test and Fisher’s exact test were used to assess the statistical relationships between categorical variables. A P-value of 0.05 was the threshold for significance.

## Results

**Fig. 2:**
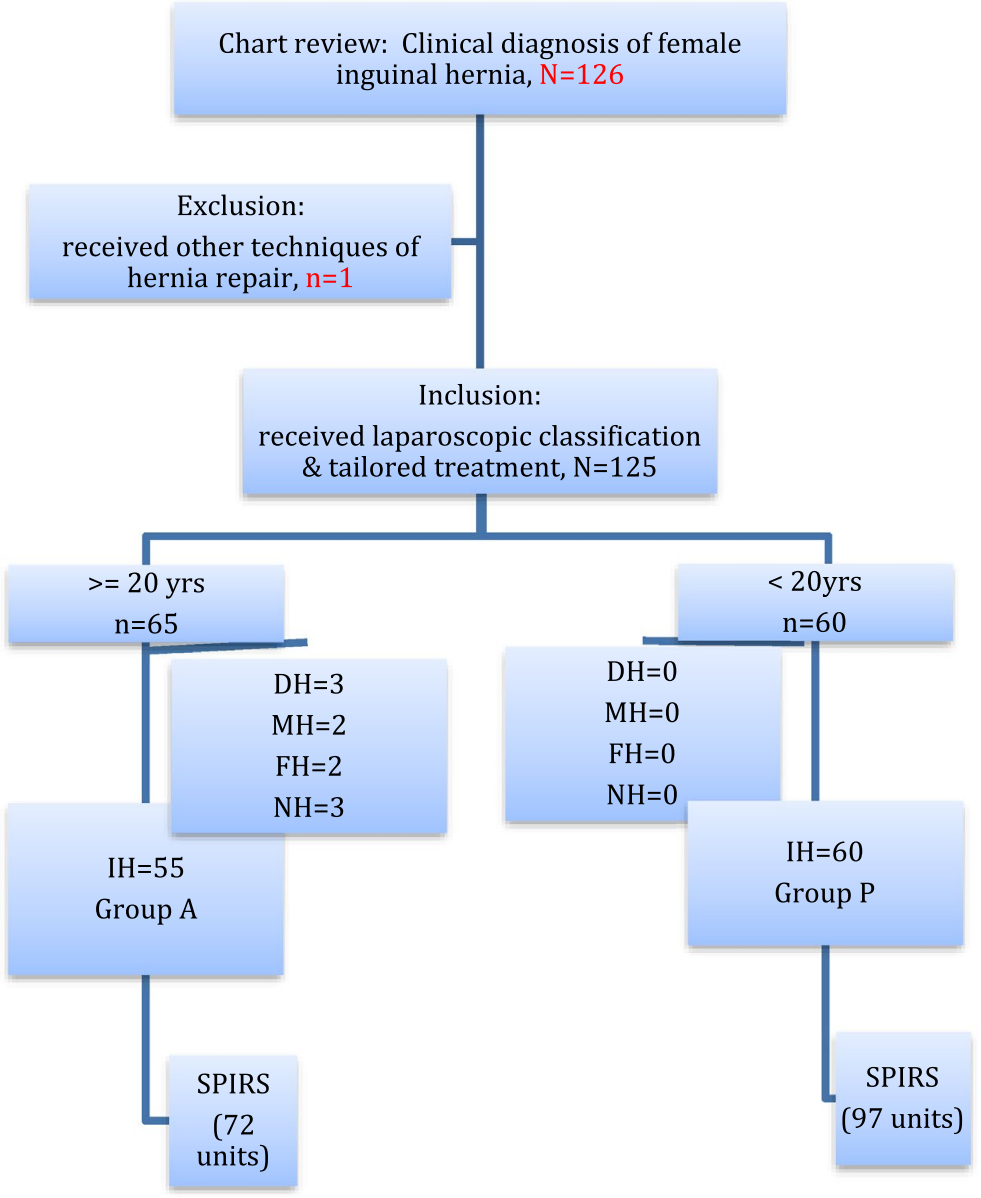
The algorithm of protocol for tailoring treatment of inguinal hernia. Female patients who were clinically diagnosed to have inguinal hernia will receive laparoscopic inspection to determine the hernia type followed by individualizing the treatment based on their subtype. DH: direct hernia, MH: mixed hernia, FH: femoral hernia, NH: non hernia, IH: indirect hernia, SPIRS: single-port laparoscopic percutaneous internal ring suture

**Fig. 3:**
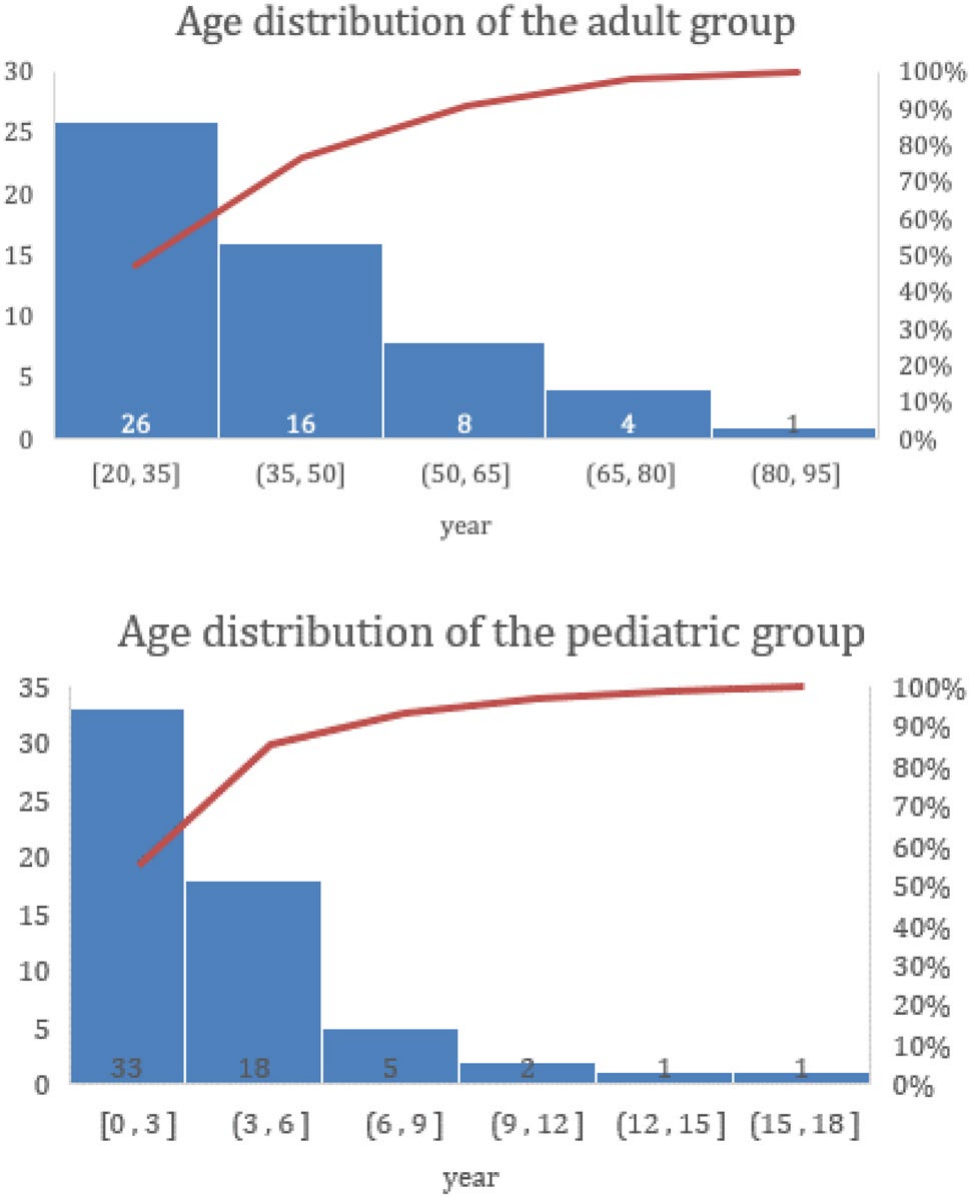
The age distribution of indirect inguinal hernia for both the adult (upper panel) and pediatric (lower panel) groups. The y-axis was patient numbers, while the x-axis was age ranged in years. The cumulated curve was expressed in red

From Oct. 2016 to May 2022, a total of 126 patients were diagnosed with inguinal hernia at our outpatient department. One 20-day-old premature baby, presenting with a left incarcerated inguinal hernia and undergoing emergency open herniorrhaphy, was excluded from this study. All the other 125 patients, including 65 adults and 60 children, received laparoscopic inspection and treatment of their hernias according to the hernia type (Fig. 2). For the adult group, there were 55 indirect type (84.6%), 3 direct type (4.6%), 2 mixed type (3.1%), 2 femoral type (3.1%), and 3 non hernias (4.6%) (Table 1). All the 60 (100%) children were indirect type. All the indirect hernias for both groups were repaired by SPIRS. The total SPIRS performed for group A and group P were 72 and 97 units respectively (*p* = *0.020*). No one needed conversion to open surgery. The median age was 38 years (range, 23–88) for group A and 3 years (range, 0.1–16) for group P. The accumulated age distribution for both groups were shown in Fig. 3.

**Table 1:** Classification of hernia types or other disease entities for the female adults

Classification	Case numbers	Op method (units)
Indirect type	55 (84.6%)	SPIRS (72)
direct type	3 (4.6%)	TEP (2), OM (1)
Mixed type	2 (3.1)	OM (2)
Femoral type	2 (3.1)	TEP (1), OM (1)
Non hernia (encystic hydrocele, lipoma, nodular fasciitis)	3 (4.6%)	OR (3)

*SPIRS* singleport laparoscopic percutaneous internal ring suture, *TEP* total extraperitoneal mesh repair, *OM* open mesh repair, *OR* open removal

The mean body weight was 54.1 ± 6.0 kg for group A and 15.9 ± 10.5 kg for group P (Table 2). The mean body mass index (BMI) for the adult group was 21.1 ± 2.4, but not available for the children group. The percentage of patients with right/left/bilateral hernias was 53/38/9 for the group A and 35/43/22 for the group P (*p* = *0.096*). The rate of contralateral patent processus vaginalis (CPPV) was 24% and 50% for group A and P respectively (*p* = *0.016*).

**Table 2:** The characteristics of both the adult (A) and pediatric (P) groups

	Group A (*N* = 55)	Group P (*N* = 60)	*p* value
Total PIRS units	72	97	< *0.01*
Median age in years (range)	38 (23,88)	3 (0.1,16)	
Body weight (kg), mean ± SD	54.1 ± 6.0	15.9 ± 10.5	
Body Mass Index, mean ± SD	21.1 ± 2.4	NA	
Right/ Left/ Bil sides (%)	53/38/9	35/43/22	*0.096*
CPPV (%)	24	50	*0.016*
Mean Op time (minutes), one/two sides	22/46	9/15	< *0.01/* < *0.01*
Overall complication rate (%): Wound infection (n/N) Recurrence (n/N) CPIP (n/N)	3/55 (5.4%) 0/55 (0%) 2/55 (3.6%) 1/55 (1.8%)	2/60 (3.3%) 2/60 (3.3%) 0/60 (0%) 0/60 (0%)	*0.106*
Follow-up months, mean ± SD	38.6 ± 15.4	42.8 ± 18.9	*0.198*

*N *total numbers of each group, *n *the numbers of each complication. The level of significance was set at a *P* value of 0.05

The average operation time for one/two sides was 22/46 min for the adults and 9/15 min for children (*p* < *0.01/0.01*). All the hospital stays lasted less than 24 h. Most of the patients resumed their daily activities in 2 days and the wounds were nearly invisible one week after operation.

There was no contralateral hernia developed during the period of follow-up.

Two children had umbilical wound infection, which resolved after oral antibiotics prescription. Two adults had recurrent hernia at the 4 and 8 months postop due to stitch loosening over the IRO. At the redo SPIRS, three stitches were applied to close the IRO. One adult patient who suffered from postoperative groin pain for 4 months and met the diagnosis of chronic postoperative inguinal pain (CPIP). As ligation of the ilioinguinal nerve during the procedure of SPIRS was highly suspected, the patient received open herniotomy and the previous stitch was removed to relieve pain. These three patients were doing well after a follow-up of over 36 months.

## Discussions

Treatment of a groin lump in females is challenging as the diversity of pathologies in this region. Although the international guidelines suggested a laparoscopic mesh repair for women’s IH, there is no universally agreed-upon standard operation for all groin hernias [13, 14]. The international guideline also recommended tailored treatment of IH according to surgeon’s experience, patient- and herniarelated characteristics and local/national resources [14].

It has been widely accepted that each hernia type has different pathogenesis [7, 15]. It was thus hypothesized that an inguinal hernia could be repaired individually according to their subtype. Historically, the preoperative classification of an IH was considered challenging and was believed to be unnecessary since it was perceived not to influence treatment decisions [8]. Although the sonographic study is relatively inexpensive and avoids radiation, a wide range of accuracy rate of sonography from 45 to 96% in classifying the IHs were reported by different institutes [16–19]. In our series, half of the adult patients ever received preoperative sonography, the accuracy rate was around 67% (unpublished data).

To customize the treatment of IHs according to their subtype, we performed a laparoscopic examination under general anesthesia and determined the adequate repair technique for each type [12]. That is, the internal ring opening is closed by using SPIRS for the indirect type, while a mesh-repaired technique is performed if a direct, mixed, or femoral type is encountered. Furthermore, if there is no abdominal wall defect found under laparoscopy, we will shift to open inguinal exploration and identify the character of this lump. We believe that accurate treatment for IHs will not only minimize unnecessary tissue trauma during operation, but also avoid inappropriate management for a groin lump, hence decreasing the postoperative recurrence rate and improving the quality of life for patients.

Consistent with our expectations, all the pediatric IHs in this series were indirect type (60/60) and received SPIRS uneventfully. Consistent with the previous literature, our series showed as high as 85% (55/65) of IHs in the female adults were indirect type and all these IIHs were successfully repaired by using SPIRS. There were 3 direct, 2 mixed, and 2 femoral hernias found during laparoscopic inspection and these cases were shifted to TEP or open mesh repair as patient’s consent (Table 1). Three cases who had an overt groin lump but without any abdominal wall defect were converted to open inguinal exploration, while one encystic hydrocele, one lipoma, and one nodular fasciitis were finally diagnosed.

As depicted in Fig. 3, our patients have covered a wide range of age distribution for both groups, from 1-month-old newborn to 88-year-old woman. Interestingly, unlike the male adults who have a peak incidence of IHs in the seventies [20], indirect IH for women seem to develop earlier in their lives as over 80% of females in this series had their IHs before the age of 6 years and 50 years for group P and A respectively. These data support the pathogenesis of an indirect IH for the female adults may also like the children, due to the non-obliteration of a congenital processus vaginalis. Thus, simple closure of the internal ring opening would be feasible for repairing the IIH of these two groups.

Reviewing the literature, incidence of the patent processus vaginalis PPV) decreased as aging [21–24]. Our series was consistent with others’ reports as the contralateral PPV (CPPV) was found to be around 50% for the pediatric and 24% for the adult group (Table 2). Although the identification and synchronized closure of the CPPV during the operation is continuous a debating issue [25], a concomitant repair was suggested by the HerniaSurge Group if CPPV was found during laparoscopy [14]. All the CPPV in this series received internal ring closure simultaneously and no metachronous hernia developed after the procedures.

It took a significantly longer op time for the adult SPIRS than children’s, one/two sides: 22/46 vs. 9/16 in minutes (*p* < *0.01/0.01*). This was due to the tissue dissection for the adult group being much more difficult. Meanwhile, 2 to 3 stitches are needed to secure the closure of an adult’s IRO.

**Fig. 4:**
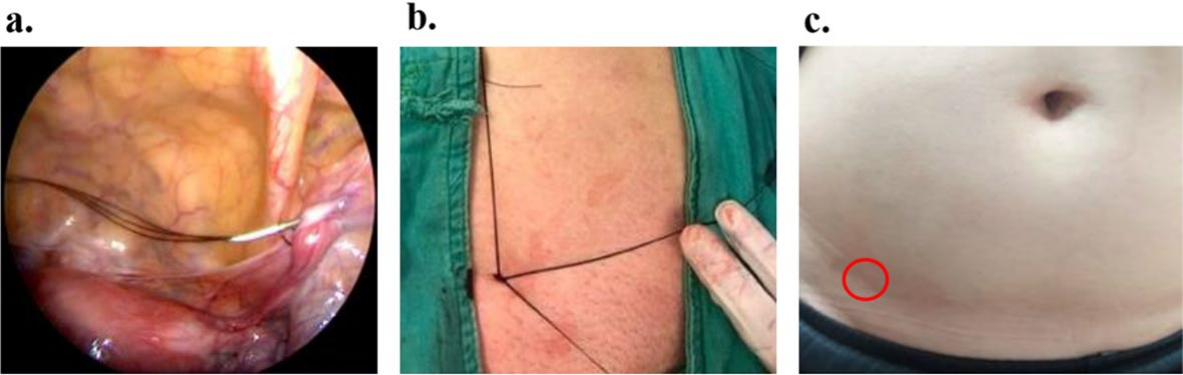
Two to three monofilament 2–0 Nylon stitches are needed to secure the closure of an adult IRO. **a** Three 2–0 Nylon were loaded simultaneously via the 18G needle. **b** The 3 non-absorbable stitches, which have encircled the IRO at the preperitoneal level, were tied sequentially to close the IRO. **c** The post-op scar was nearly invisible one-week post-op

There was no recurrence for the pediatric group. However, two adult cases recurred at the 4th and 8th month post-op with a recurrent rate of 3.6%. At the redo surgery, loosening of the previous stitch was noted and the IRO was reclosed using three non-absorbable stitches. These 2 recurrent cases were among the first 20 adult patients of this series, in whom only one stitch was used to close the IRO. We suggested that the intraabdominal pressure in the adult group was much higher than in the children. After that, for the remaining 35 adult patients, we applied 2–3 non-absorbable stitches over the IRO and no further recurrent cases noted (Fig. 4).

We found that postoperative inguinal pain was more prominent for the adults than children. This may be due to the well development of the abdominal wall nerves for the adults. Fortunately, most of the patients resumed their daily life after 2 or 3 days of oral NSAID. The chronic postoperative inguinal pain (CPIP), persistent inguinal pain over 3 months after hernia surgery, is another important issue. Although incidence of CPIP was estimated to be around 10% in the adult cases [26], CPIP after the pediatric PIRS has never been reported [27]. As expected, there was no chronic pain found in our pediatric group. However, a 34-year-old woman who suffered from CPIP over the region of labia majora for 4 months after surgery. As a mechanical stitch ligation over the ilioinguinal nerve was highly suspected, an elective open inguinal exploration was scheduled. During the surgery, the previous suture stitch was removed and simple high ligation of hernia sac performed. The patient’s CPIP resolved completely. This implies that the CPIP induced by this minimally invasive technique could be a reversible condition after the relief of suture stitch.

Patients’ history of previous caesarean section or pelvic endometriosis had not become an obstacle to the creation of pneumoperitoneum when we used direct trocar insertion technique [28, 29]. Because the 3-mm, sharp-ended trocar enabled us to make a quick access into the abdominal cavity and then safely create pneumoperitoneum under direct vision provided by laparoscope. In our experience, only one adult patient with an umbilical scar due to previous laparoscopic colectomy needed an open method (Hasson) to access the abdominal cavity. Until now, there have been no complications developed during the creation of pneumoperitoneum by using this direct puncture method.

Based on the hypothesis that the adult indirect IH is due to the non-obliteration of patent processus vaginalis, we have successfully applied the technique of SPIRS for the repair of IIH in female adults. Our data showed the outcomes are comparable between the pediatric and adult group by using this minimal invasive technique.

Compared to the modern tension-free mesh repair techniques, the SPIRS avoids the complications of synthetic mesh, including migration, foreign body sensation, and mesh infection. Furthermore, due to the minimal tissue invasion, none of our patients developed seroma/hematoma after SPIRS. All the patients who received SPIRS had their hospital stay less than 24 h and resumed their daily activity in 2–3 days. The op scars were nearly invisible one week post op (Fig. 4c). However, more case accumulation is needed to evaluate the long-term results of SPIRS in female adults.

To our knowledge, this is the first report that customized the treatment of female IHs according to their subtype and applied the technique of SPIRS to the treatment of female IIH. Although we have achieved a very excellent outcome, there are some cons for this tailored procedure. First, patients have to undergo general anesthesia, increasing risk for the patients with premature, old age or have cardiopulmonary diseases. In fact, this is why the only one excluded case who did not choose this technique initially. Second, one more step, and thus extra time, is needed to create pneumoperitoneum for the laparoscopic inspection. The pneumoperitoneum per se may also be a relative contraindication for patients who experienced abdominal surgeries. Third, the surgeons need to get familiar with at least two techniques, *i.e.* SPIRS and TEP or Lichtenstein, to cope with the complexity of female IHs. More prospective studies should be conducted to evaluate the long-term results of this novel management for the female IHs.

## Conclusion

Our results support that the pathogenesis of an adult indirect inguinal hernia is due to the nonobliteration of a congenital processus vaginalis. The tailored approach of treating female IIHs using the single-port laparoscopic percutaneous internal ring suture appears promising and could serve as an alternative for managing female IHs. However, further investigations comparing this method to traditional techniques are warranted.

## Data Availability

The datasets generated and analyzed during the current study are available from the corresponding author on reasonable request.
